# Changes in the bacterial communities of *Harmonia axyridis* (Coleoptera: Coccinellidae) in response to long-term cold storage and progressive loss of egg viability in cold-stored beetles

**DOI:** 10.3389/fmicb.2024.1276668

**Published:** 2024-03-12

**Authors:** Yuanxing Sun, Yanan Hao, Senshan Wang, Xinling Chen

**Affiliations:** Biocontrol Engineering Laboratory of Crop Diseases and Pests of Gansu Province, College of Plant Protection, Gansu Agricultural University, Lanzhou, China

**Keywords:** cold storage, reproductive tracts, bacteria, *Harmonia axyridis*, egg viability

## Abstract

Bacteria have a profound influence on life history and reproduction of numerous insects, while the associations between hosts and bacteria are substantially influenced by environmental pressures. Cold storage is crucial for extending the shelf life of insects used as tools for biological control, but mostly causes detrimental effects. In this study, we observed a great decrease in egg hatch rate of cold-stored *Harmonia axyridis* during the later oviposition periods. Furthermore, most eggs produced by their F1 offspring exhibited complete loss of hatchability. We hypothesized that long-term exposure to cold may greatly alter the bacterial community within the reproductive tracts of *H. axyridis*, which may be an important factor contributing to the loss of egg viability. Through sequencing of the 16S rRNA gene, we discovered considerable changes in the bacterial structure within the reproductive tracts of female cold-stored beetles (LCS_F) compared to non-stored beetles (Control_F), with a notable increase in unclassified_f_Enterobacteriaceae in LCS_F. Furthermore, in accordance with the change of egg hatchability, we observed a slight variation in the microbial community of eggs produced by cold-stored beetles in early (Egg_E) and later (Egg_L) oviposition periods as well as in eggs produced by their F1 offspring (Egg_F1). Functional predictions of the microbial communities revealed a significant decrease in the relative abundance of substance dependence pathway in LCS_F. Moreover, this pathway exhibited relatively lower abundance levels in both Egg_L and Egg_F1 compared to Egg_E. These findings validate that long-term cold storage can greatly modify the bacterial composition within *H. axyridis*, thereby expanding our understanding of the intricate bacteria-insect host interactions.

## Introduction

Studying microorganisms is a fundamental aspect to understand insect biology (Dillon and Dillon, 2004). The insect-bacteria association constitutes a common symbiotic interaction and is known to regularly alter the life history of the host (Moran and Baumann, 2000; Oliver et al., 2010; Bennett and Moran, 2013; Salem et al., 2015). While extensive research has been conducted on gut bacteria and their functions, our understanding of the microbiome within the reproductive tract remains comparatively limited (Zhao et al., 2022). Remarkably, bacteria residing within or transmitted through reproductive tissues exert profound influences on host reproduction, health, and evolutionary processes (Perlmutter and Bordenstein, 2020). In particular, copulation exposes both male and female to their partner’s gonad-associated bacteria, and dysbiosis in either sex can affect the other, significantly impairing their joint and individual fitness (Otti, 2015; Bellinvia et al., 2020a). Additionally, the reproductive system has been identified as a potential route for transgenerational bacterial inoculation (Lavy et al., 2020), thereby enabling eggs to acquire bacteria through transovarial and/or parental transmission routes (Perlmutter and Bordenstein, 2020).

For a given insect species, its microbiomes are usually dynamic and influenced by a series of environmental factors, including diet and climate (Bellinvia et al., 2020b). Extensive research has been conducted on the impact of dietary factors. For example, *Aphis fabae* Scopoli feeding *Lamium purpureum* L. attained high densities of *Regiella insecticola* and *Hamiltonella defensa* compared to that consuming *Vicia faba* L. (Chandler et al., 2008). Crickets fed with a chemically defined diet displayed a predominant Bacteroidetes in the gut, representing 74% ± 10% of the total bacteria, almost twice as much compared to the cat chow-fed crickets (Ng et al., 2018). Thermal conditions are also important factors determining the insect-symbiont interactions; furthermore, many microbes themselves are sensitive to thermal variability. Previous studies have extensively explored the effects of heat stress (Tougeron and Iltis, 2022). However, there is limited reporting on the variability of bacteria and host-bacteria associations under cold conditions.

*Harmonia axyridis* (Pallas) is an important natural enemy of aphids and is also recognized as a notorious invasive species (Brown et al., 2011; Camacho-Cervantes et al., 2017). This ladybird beetle harbors a myriad of endosymbiotic microbes throughout life stages, with higher richness and diversity of bacterial community observed in eggs, followed by adults and pupae (Du et al., 2022). In recent years, several studies have been conducted to reveal the response of *H. axyridis*’s bacterial community to changes in food source or pesticide pressure. Huang et al. (2022) found that the diversity of gut microbiota was greatly affected by food sources (*Acyrthosiphon pisum* Harris, *Diaphorina citri* Kuwayama, and artificial diets). For example, the Enterobacteriaceae exhibited the highest abundance on diet with *D. citri*, whereas the Staphylococcaceae demonstrated the highest abundance on artificial diet. Wang et al. (2022) observed an increase in intestinal microorganism abundance when conspecific eggs plus aphids were consumed, while a decrease was noted when *Coccinella septempunctata* L. eggs plus aphids were ingested. In addition, Luo et al. (2023) observed a significant increase in the abundance of *Enterococcus*, *Serratia*, and *Enterobacter* following consumption of azadirachtin-treated *Spodoptera frugiperda* (J.E. Smith) larvae.

As a biological control agent, *H. axyridis* is widely used to control many agricultural and forest pests due to its high fertility and strong predatory capacity (Kenis et al., 2020). However, low-temperature storage logistics have emerged as a critical impediment to the large-scale application of this beetle in pest management (Wu et al., 2016; Tang et al., 2017). In our previous study involving laboratory-reared adults, a 120-day cold storage resulted in a significant decline in egg hatch rate starting from day 6, eventually reaching near-zero on days 13–15. In coccinellids, the manipulation of host reproduction by bacteria (e.g., *Spiroplasma* and *Wolbachia*) has been extensively documented (Hurst and Jiggins, 2000; Elnagdy et al., 2011, 2013). In this study, we aimed to investigate the impact of long-term cold storage on the bacterial composition of *H. axyridis* while inducing a decrease in egg hatchability. To achieve this objective, we analyzed the bacterial composition in the reproductive tracts of both cold-stored and non-stored adults. Additionally, we examined the changes in bacterial communinty in eggs produced by cold-stored beetles during early and later oviposition periods as well as in eggs produced by their F1 offspring with different hatchabilities. Specifically, adult beetles were subjected to storage at a temperature of 5°C for up to 120 days to induce more severe cold stress compared to our previous study where storage was conducted at 6°C (Sun et al., 2022). The findings of this study will broaden our understanding of the impacts of environmental factors on bacterial composition and on bacteria-insect host interactions. Additionally, these results will aid in elucidating the mechanisms underlying the sublethal effects induced by cold storage on natural enemies.

## Materials and methods

### Insects

Three pairs of *H. axyridis* f. *succinea* were collected from the field of the broad bean *V. faba* at Gansu Agricultural University, Lanzhou, Gansu Province, China in early spring 2021. In our laboratory, the beetles were continuously reared using the green morph pea aphid *A. pisum*. The aphid colony was established in nylon cages (45 × 45 × 45 cm) containing broad bean seedlings. All insects were maintained under controlled conditions in an insectary (25 ± 1°C with 60% RH, and a photoperiod 14: 10 L: D).

To provide sufficient adults for cold storage, newly hatched *H. axyridis* larvae were reared in specially designed containers (95.38 cm^3^) at a density of 32 larvae per container (Sun et al., 2021) during the period from October to November 2022. The larvae received a daily refreshed *A. pisum* and their development stages were monitored once a day. The newly emerged adults were utilized for subsequent experimental procedures.

### Cold storage treatments and preparation of reproductive tract samples

Before subjecting *H. axyridis* adults to cold storage, they underwent a period of pre-storage feeding and cold acclimation following the methods described in our previous studies (Sun et al., 2019, 2022). In brief, female and male adults were separately reared in plastic Petri dishes (9 cm in diameter) at a density of 6–8 adults per dish. They were kept in an incubator (RDN-300D-5, Ningbo Southeast Instrument Co., LTD, Zhejiang, China) at 25 ± 1°C with 60% RH and a photoperiod 14: 10 L: D. Each dish was provided with three diet patches (approximately 0.2 mL each), which were refreshed every other day. Additionally, two cotton balls immersed with distilled water were supplied for water provision. Ten days later, the adults were transferred to another incubator and kept at 15 ± 1°C for 2 days of cold acclimation. Subsequently, the 13-day old adults were stored at a temperature of 5°C within a refrigerator (under full darkness condition) for long-term cold storage of 120 days (LCS group). After storage, the live adults (42 females and 50 males) were quickly killed in liquid nitrogen and stored in a −80°C ultra low-temperature refrigerator until used. Meanwhile, non-stored beetles (31 females and 40 males), which underwent identical pre-storage feeding and cold acclimation periods, were also stored at −80°C serving as the control group.

The reproductive tracts of adult beetles from the LCS group and those from the control group were dissected according to the protocols outlined by Huang et al. (2022) and Du et al. (2022). The adult surface was sterilized by immersion in 70% absolute ethanol for 3 min and rinsed three times in sterile phosphate-buffered saline. The animals were then dissected aseptically in a sterile phosphate-buffered saline solution. 70% absolute ethanol and flame were used for the sterilization of the workstation and dissection tools, respectively. Excised reproductive tracts were collected in 1.5-ml sterile centrifuge tubes, and female and male samples of LCS and control group were named LCS_F, Control_F, LCS_M, and Control_M, respectively. Each sample had three replicates.

### Reproductive performances of cold-stored beetles and preparation of egg samples

**Figure 1:**
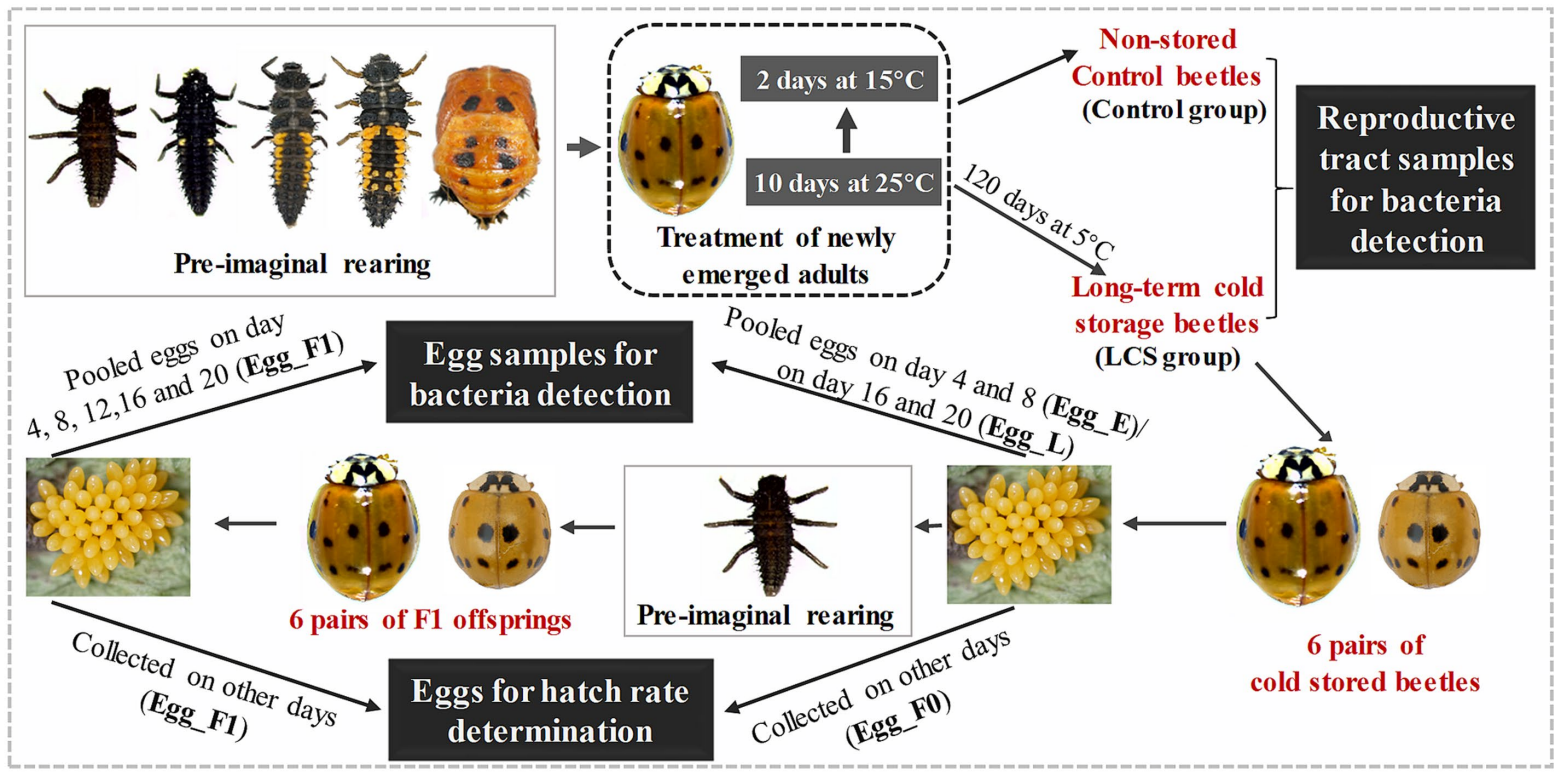
An overview of the experimental design. Briefly, newly emerged adults of *Harmonia axyridis* were reared on an artificial diet at 25°C for 10 days, followed by exposure to 15°C for 2 days. After that, the adults were divided into two groups: one group was directly used for obtaining reproductive tract samples for bacteria detection (Control group), while the other group was stored at 5°C for 120 days and then used for obtaining reproductive samples (LCS group). Additionaly, 6 pairs of cold stored beetles as well as their F1 offsprings were reared to collect eggs either for bacteria detection or hatch rate determination. Specifically, pooled eggs produced by cold stored beetles on day 4 and 8 (Egg_E) as well as on day 16 and 20 (Egg_L), along with pooled eggs produced by F1 offsprings on day 4, 8, 12, 16, and 20 (Egg_F1), were used for bacteria detection. Eggs produced on other days by cold stored beetles (Egg_F0) and their F1 offsprings (Egg_F1) were used for egg hatch rate determination. The beetles were reared with pea aphid throughout the whole experiment, except for treatment of newly emerged adults (as shown in dashed dark box). The beetles within the gray box represent key developmental stages of *H. axyridis* during pre-imaginal rearing, while several stages were omitted in F1 offspring.

To further monitor changes in the egg bacterial communities associated with variable viability in cold-stored beetles, eight pairs LCS group beetles were transferred to 25 ± 1°C and reared with *A. pisum*. Each pair were placed in a plastic Petri dish (9 cm in diameter) and their egg production was monitored once a day. During the experiment, unfortunate mortality occurred for one female and one male beetle from different pairs, resulting in the exclusion of these two pairs from analysis. When egg production started, eggs produced over the subsequent 20 days were collected. Eggs produced on day 4, 8, 12, 16, and 20 were stored at −80°C for bacterial detection (at each time point, eggs from the six pairs were pooled together), while those produced on other periods were incubated at 25°C to determine hatchability (Egg_F0, eggs from each pair were kept separately in individual plastic petri dishes). Furthermore, 15 neonate larvae emerged from the eggs produced on days 9–10 were consistently reared with *A. pisum*. A total of six pairs of adult beetles (F1 offspring of cold-stored beetles) were obtained and their eggs were collected for bacterial detection or determination of egg viability (Egg_F1) using the same methods as described in their parental generation (F0) (Figure 1).

We found that eggs produced by cold-stored beetles on days 1–3 and 5–7 had relatively high hatch rates, whereas those produced on days 13–15 and 17–19 showed extremely low hatchability. Therefore, we combined eggs collected on day 4 and 8 to represent early stage samples (Egg_E), while pooled eggs from day 16 and 20 were used as late stage samples (Egg_L). Furthermore, the F1 offspring of cold-stored beetles consistently yielded eggs with low hatch rates. Thus, pooled eggs from day 8, 12, 16, and 20 were used to represent the F1 generation samples (Egg_F1). Following methods described by Du et al. (2022), the egg samples, 100 in each replicate, were sterilized by washing them with 70% ethanol for 30 s, followed by rinsing thrice with phosphate-buffered saline solution. Each sample had three replicates.

### DNA extraction and PCR amplification

Total bacterial genomic DNA was extracted from ladybird reproductive tracts and eggs using the TIANamp Genomic DNA Kit (TIANGEN Biotech, Beijing, China). The concentration and the integrity of extracted DNA were quantified using the NanoDrop 2000C spectrophotometer (Thermo Scientific, United States) and 1% (w/v) agarose gel electrophoresis, respectively. Then the V3-V4 region of the bacterial 16S rRNA gene was amplified using primer 338F 5-ACTCCTACGGGAGGCAGCAG-3 and 806R 5-GGACTACHVGGGTWTCTAAT-3 (Chu et al., 2015) using a GeneAmp 9700 thermocycler PCR system (ABI, United States). The PCR components included 10 ng of template DNA, 4 μL of 5 × FastPfu buffer, 2 μL of 2.5 mM dNTPs, 0.4 μL of FastPfu polymerase, 0.8 μL (5 μM) of each primer, and nuclease-free water. The PCR was run at 95°C for 3 min, 29 cycles at 95°C for 30 s, 53°C for 30 s, and 72°C for 45 s with a final extension of 10 min at 72°C. The PCR products were visualized on 2% (w/v) agarose gels after amplification.

### Illumina MiSeq platform sequencing

The PCR products were excised from 2% (w/v) agarose gels, purified using the AxyPrep DNA Gel Extraction Kit (Axygen Biosciences, Union City, CA, United States), and quantified using QuantiFluor^™^-ST (Promega, United States) according to the manufacturer’s protocol. The purified amplicons were pooled in equimolar amounts, and sequencing was conducted on an Illumina MiSeq PE300 platform at Majorbio Co., Ltd. (Shanghai, China).

### Data analysis

Statistical analysis was performed using R software (R Core Team, 2020). The non-parametric Kruskal-Wallis test was used for the analysis of the three-day average egg hatch rates of Egg_F0 and Egg_F1, respectively. When a significant difference was detected (*p* < 0.05), the Mann–Whitney U-test with Bonferroni correction was used for multiple comparisons (Ghazy et al., 2014). The independent sample *t* test was used to compare the differences between Egg_F0 and Egg_F1.

For bacteria community, raw sequence reads were demultiplexed, quality-filtered using Trimmomatic, and merged using FLASH (Mago and Salzberg, 2011). Sequence analysis was performed with Uparse software. Sequences with ≥97% similarity were assigned to the same OTUs (Edgar, 2013). Subsequently, all effective 16S rRNA gene sequences were aligned against the SILVA (SSU132) 16S rRNA database. Then, through the Ribosomal Database Project Classifier (Quast et al., 2012), the species classification information corresponding to each OTU was obtained using a 70% confidence threshold. Using MOTHUR, alpha diversity is applied in analyzing complexity of species diversity for a sample through 3 indices, including Shannon (community diversity), Chao (community richness), and Good’s coverage (sequencing depth) (Schloss et al., 2009; Caporaso et al., 2010). Differences in alpha diversity between groups were compared using a one-way ANOVA for normally distributed data; non-normally distributed samples were subjected to the Kruskal-Wallis test for comparison between groups. Beta diversity analysis was used to evaluate differences of samples in species complexity using Unweighted and Weighted UniFrac distance metrics (Lozupone and Knight, 2005) principal coordinates analysis (PCoA). The significance of differentiation of microbiota structure among groups was assessed by adonis with 999 permutations (Oksanen et al., 2019). The venn diagram was implemented using the Venn Diagram R package. To detect potential biomarkers, the linear discriminant analysis (LDA) effect size (LEfSe) method was used based on a normalized relative abundance matrix. The LEfSe method uses the Kruskal-Wallis test to identify features with significant differences among different groups of *H. axyridis* and performs LDA to evaluate the effect size of each feature (Segata et al., 2011). In addition, Phylogenetic Investigation of Communities by Reconstruction of Unobserved States (PICRUSt) was used to predict the Kyoto Encyclopedia of Genes and Genomes (KEGG) category and obtain the levels of metabolic pathway information (Langille et al., 2013).

## Results

### Reproductive performances of long-term cold-stored *Harmonia axyridis* adults

**Figure 2:**
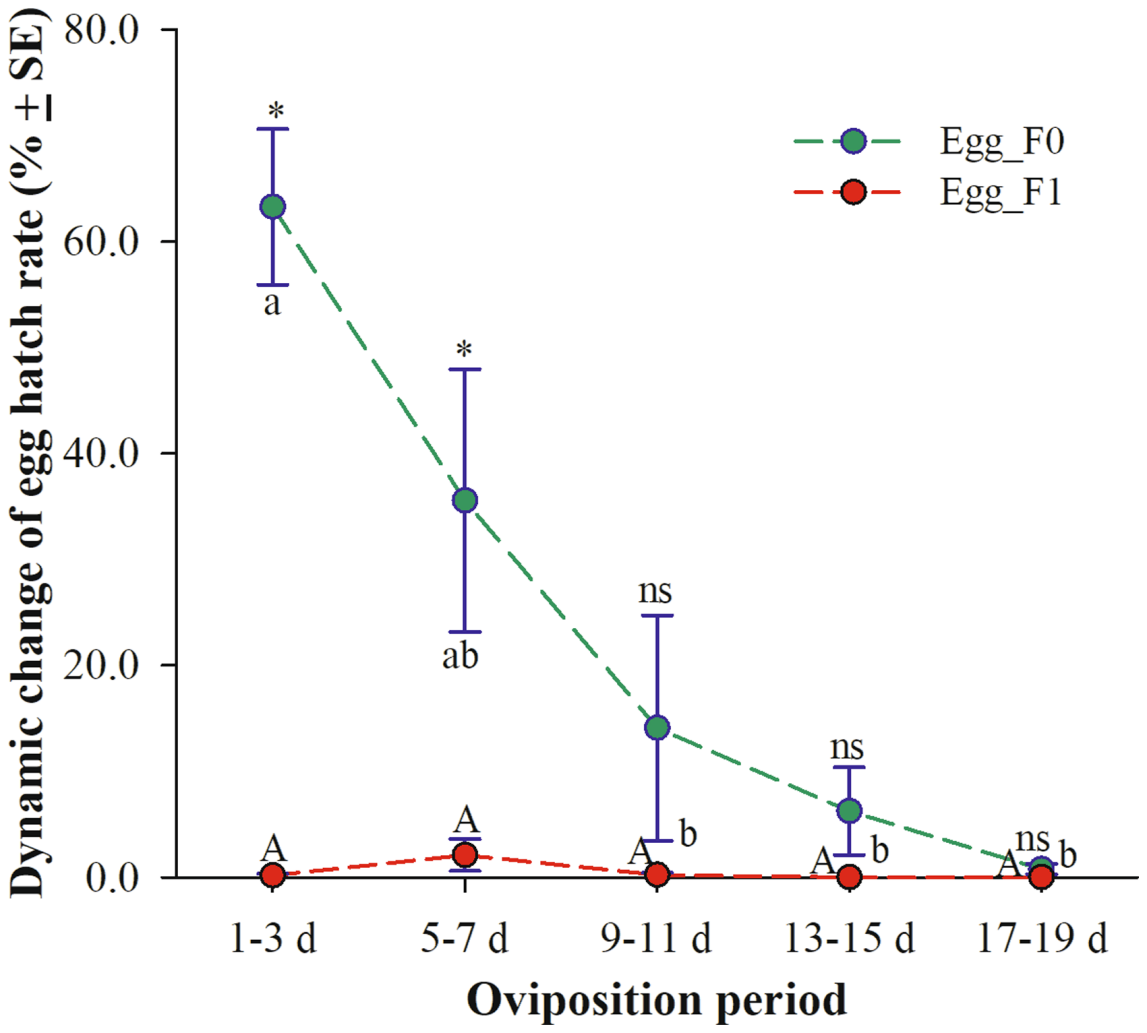
Dynamic changes of three-day average hatch rates of eggs produced by cold-stored beetles (Egg_F0) and their F1 offspring (Egg_F1) over 20 days. Different lowercase letters represent a significant difference among different oviposition periods of Egg_F0 (*p* < 0.05), while same uppercase letter represents no significant difference among different oviposition periods of Egg_F1 (*p* > 0.05). Asterisk represents a significant difference between Egg_F0 and Egg_F1 (*p* < 0.05), while ns means no significant difference (*p* > 0.05).

Egg_F0, produced by cold-stored beetles, exhibited a significant decrease in three-day average hatch rates as the oviposition period increased. Specifically, the hatch rates were above 35.6% on days 1–3 and 5–7 but dropped below 7% on days 13–15 (6.2%) and 17–19 (0.7%) (χ^2^ = 14.653, df = 4, *p* = 0.005). However, the hatch rates of Egg_F1 remained consistently low throughout the entire oviposition period of 20 days with only a fraction of viable eggs observed within the first 10 days (still less than 2.0%) (χ^2^ = 7.203, df = 4, *p* = 0.126). Meanwhile, it was found that the egg hatch rates on days 1–3 and 5–7 were significantly lower for Egg_F1 compared to those for Egg_F0 (1–3 d: *t =* 8.600, df = 10, *p* < 0.001; 5–7 d: *t =* 2.680, df = 10, *p* = 0.023) (Figure 2).

### Microbial community of adult reproductive tracts under cold storage

**Figure 3:**
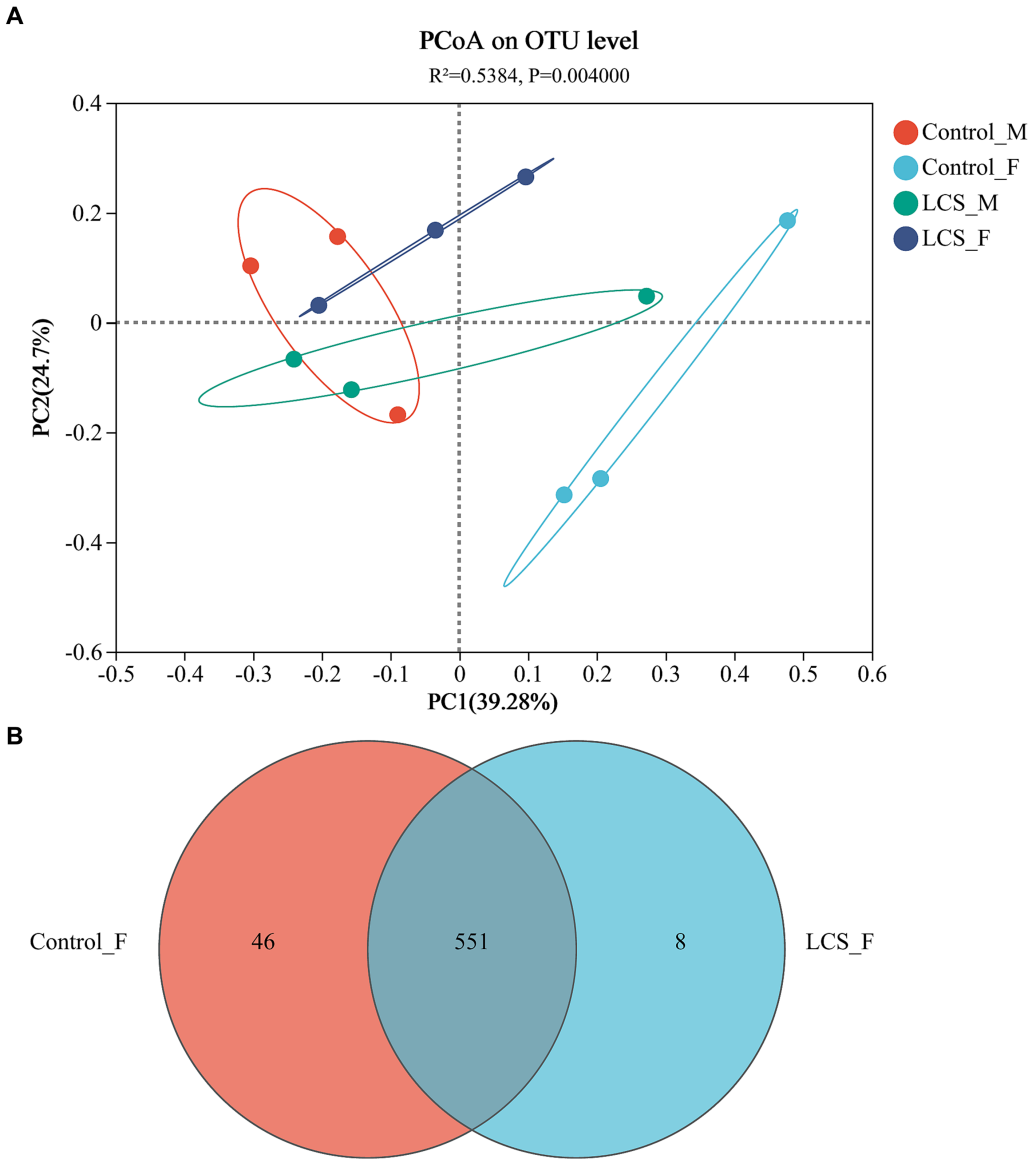
Bacterial communities in the reproductive tracts of cold-stored adults of *Harmonia axyridis*. **(A)** Weighted UniFrac-based PCoA plots of bacterial communities. **(B)** Number of OTUs of Control_F and LCS_F. Control_F and Control_M represents the female and male reproductive tracts of control non-stored beetles, respectively; LCS_F and LCS_M represents the female and male reproductive tracts of long-term cold stored beetles, respectively.

The PCoA based on Unifrac distances showed 39.28 and 24.70% variability in the data, respectively. Relatively short distances were detected between long-term cold-stored male beetles (LCS_M) and control non-stored male beetles (Control_M). However, a significant separation was observed between long-term cold-stored female beetles (LCS_F) and control non-stored female beetles (Control_F) (*p* = 0.004), indicating a substantial impact of cold storage on the microbial communities within the female reproductive tract (Figure 3A). Moreover, Control_F had more OTUs (597) and a greater proportion (7.7%) of unique ones as compared to LCS_F (559 OTUs, 1.4% of unique ones) (Figure 3B). All samples had a Good coverage index greater than 0.99, indicating that they were sequenced at a reasonable depth and sequenced adequately (Supplementary Figure S1A). In addition, the Shannon and Chao indices for community diversity and richness demonstrated no statistically significant difference among the four reproductive tract samples (Supplementary Figures S1B,C).

**Figure 4:**
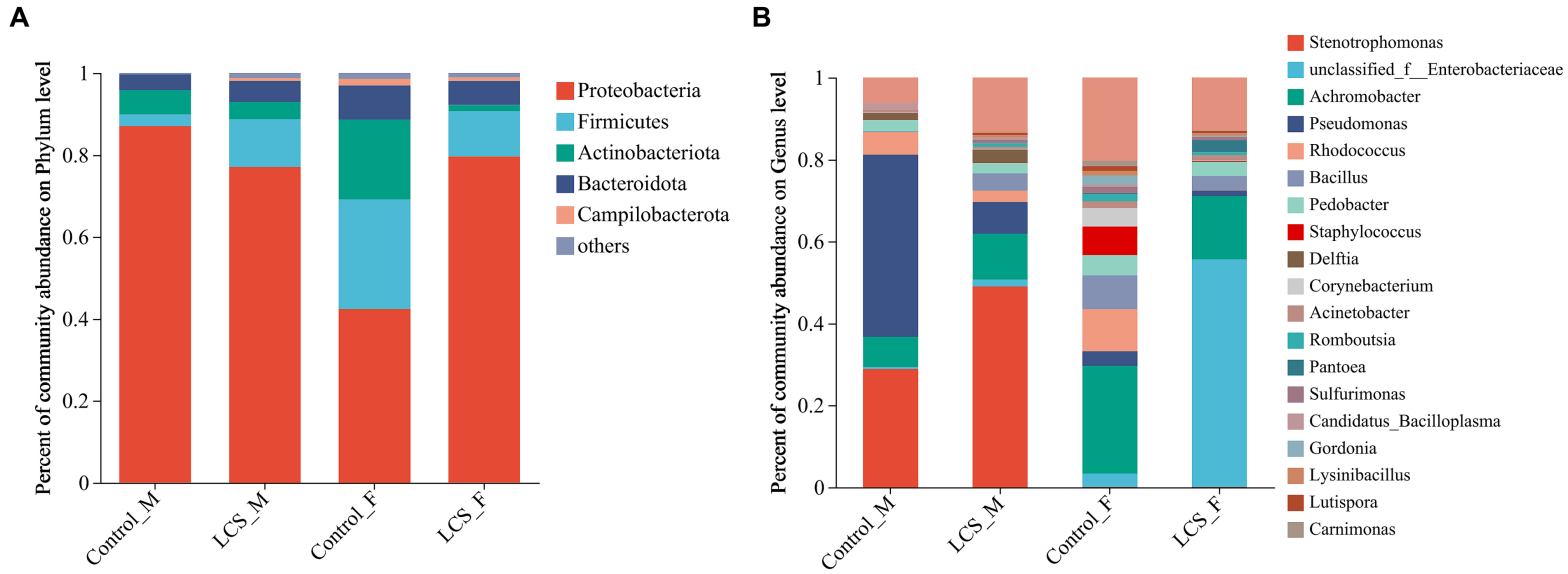
Bacterial community composition at the level of **(A)**, phylum, and **(B)**, genus in the reproductive tracts of cold-stored adults of *Harmonia axyridis*. OTUs <0.01% of the average relative abundance in groups are summarized as “others”.

Normalized sequences were aligned against the SILVA database and clustered into different taxonomic levels using a 70% threshold. At the phylum level, the top phylum with the highest relative abundance in all samples was Proteobacteria, followed by Firmicutes, Actinobacteria, and Bacteroidota. In particular, LCS_F displayed a relatively higher proportion of Proteobacteria (79.64%) compared to Control_F (42.37%); in contrast, LCS_F exhibited lower relative abundances of Firmicutes and Actinobacteriota (11.07, 1.50%) compared to Control_F (26.81, 19.48%) (Figure 4A).

At the genus level, unclassified_f_Enterobacteriaceae vastly increased in LCS_F (55.58%) compared to Control_F (3.27%). In contrast, we observed a decrease in the relative abundance of other genera such as *Achromobacter*, *Pseudomonas*, *Rhodococcus*, and *Staphylococcus* in the female tracts of cold-stored beetles. Regarding to the male reproductive tracts, *Pseudomonas* exhibited a substantial decrease in LCS_M (7.75%) compared to Control_M (44.54%), while an increased relative abundance of *Stenotrophomonas* was detected in LCS_M (49.02% versus 28.89% in Control_M) (Figure 4B).

**Figure 5:**
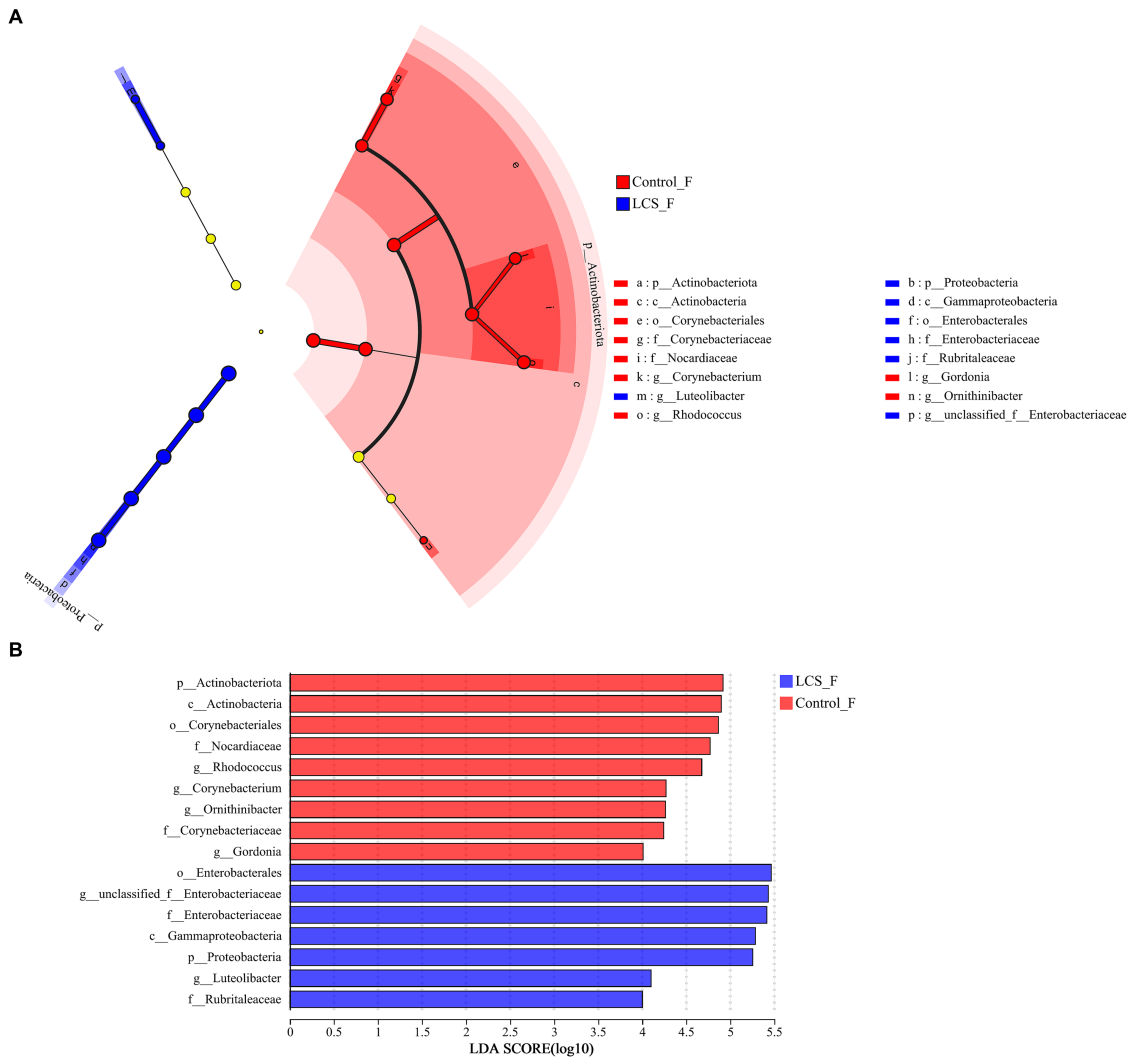
Linear discriminant analysis effect size (LEfSe) and the cladogram identifying the significant differentially abundant bacterial taxa within the reproductive tracts of Control_F and LCS_F. **(A)** Linear discriminant analysis effect size. **(B)** Taxonomic cladogram derived from LEfSe with an LDA score > 4.0.

LEfSe was used to identify key phylogeny types that differ in microbial abundance between LCS_F and Control_F. At the genus level, six bacterial clades showed statistically significant differences with an LDA threshold of 4.0 and *p* < 0.05. Control_F had a preponderance of *Rhodococcus* and *Gordonia* (Nocardiaceae), and *Corynebacterium* (Corynebacteriaceae), while LCS_F showed unclassified_f_Enterobacteriaceae (Figure 5).

### Microbial community of eggs produced by cold-stored beetles along with progressive loss of hatchability

**Figure 6:**
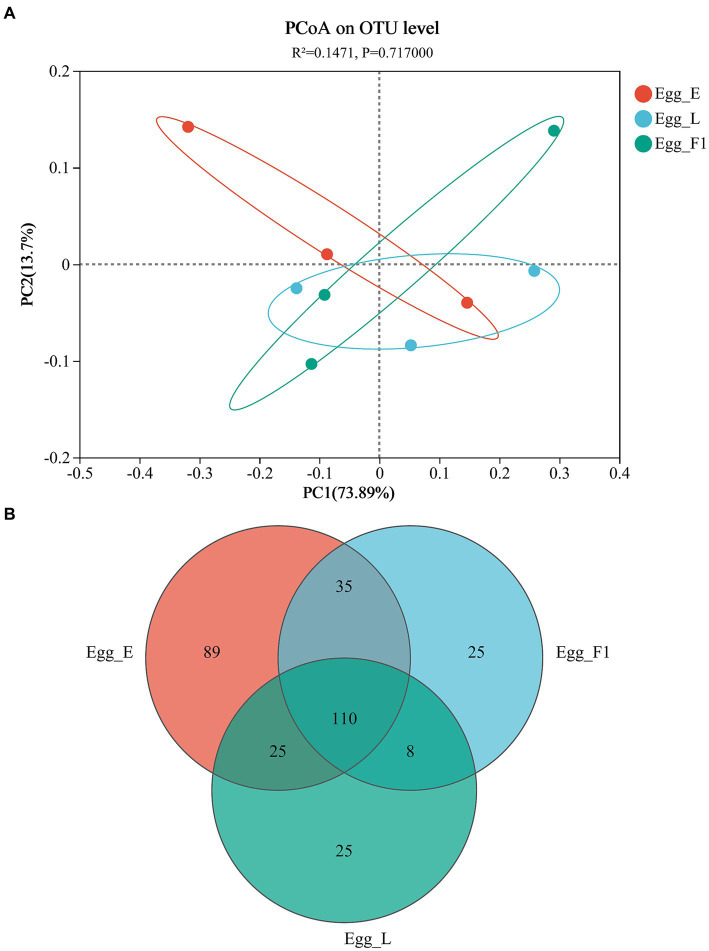
Bacterial communities in eggs of cold-stored adults of *Harmonia axyridis* and their F1 offspring. **(A)** Weighted UniFrac-based PCoA plots of bacterial communities. **(B)** Number of OTUs. Egg_E and Egg_L represents the early stage and late stage eggs of long-term cold-stored beetles, respectively, while Egg_F1 represents the eggs produced by F1 offspring adults of long-term cold stored beetles.

The PCoA based on Unifrac distances revealed a variability of 73.89 and 13.70% in the data, respectively. No significant difference was detected in the bacterial community structures among the three egg samples (*p* = 0.717; Figure 6A). In addition, Shannon and Chao indices for community diversity and richness also showed no significant difference among the three egg samples (Supplementary Figures S1B,C). However, Egg_L and Egg_F1 had fewer OTUs (168 and 178, respectively) and lower proportions (14.9 and 14.0%) of unique ones as compared to Egg_E (259 OTUs, 34.4% of unique ones) (Figure 6B).

**Figure 7:**
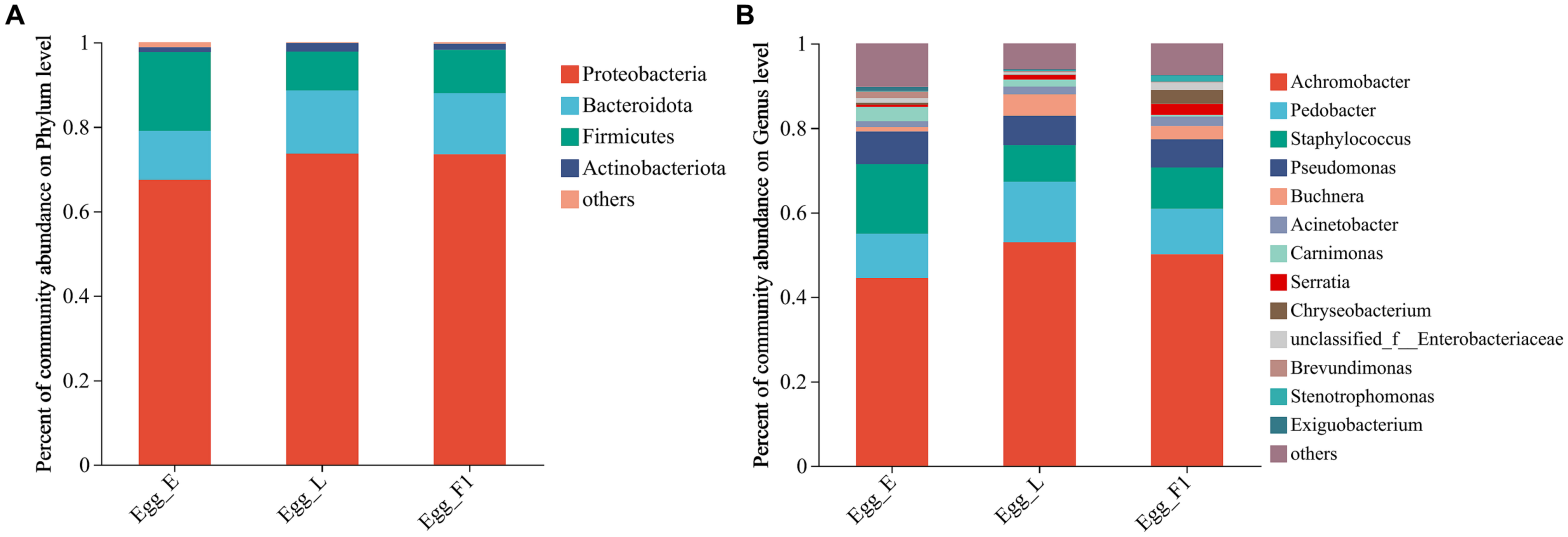
Bacterial community composition at the level of **(A)**, phylum, and **(B)**, genus within eggs. OTUs <0.01% of the average relative abundance in groups are summarized as “others”.

At the phylum level, Proteobacteria exhibited the highest relative abundance in all egg samples, followed by Firmicutes and Bacteroidota. In particular, the relative abundance of Firmicutes was comparatively higher in Egg_E (18.61%) than in Egg_L (9.20%) and Egg_F1 (10.28%) (Figure 7A). At the genus level, higher relative abundances of *Staphylococcus* and *Carnimonas* were detected in Egg_E compared to Egg_L and Egg_F1. In contrast, a higher abundance of *Buchnera* and *Serratia* was detected in Egg_L and Egg_F1 compared to Egg_E (Figure 7B). The most representative bacterial genera identified in Egg_E were *Dechloromonas* and *Lactobacillus*, while *Comamonas*, *Methyloversatilis*, *Pesudacidovorax*, and *Bacteriovorax* were observed in Egg_F1. No representative bacterial genera was detected in Egg_L when compared to Egg_E and Egg_F1 (Supplementary Figure S2).

### Functional prediction of microbiota

**Figure 8:**
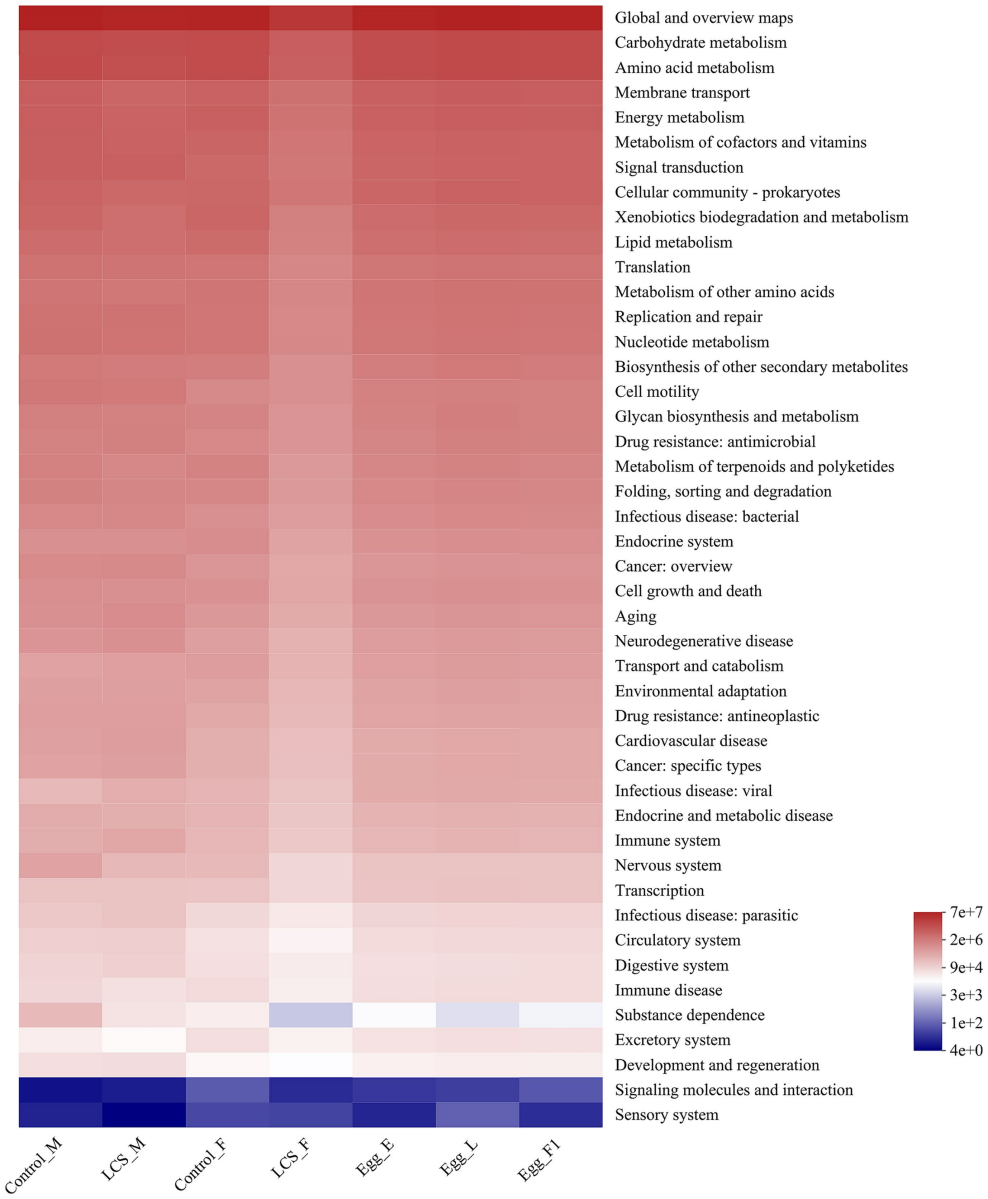
Inferred functions of the bacterial communities associated with the effects of long-term cold storage on *Harmonia axyridis*. All of the predicted Kyoto Encyclopedia of Genes and Genomes (KEGG) metabolic pathways are shown at the second hierarchical level.

Using PICRUSt, we identified significant differences in the functional potentials of bacterial community composition across the seven samples. Regarding the richness of main pathways at level 2, most pathways exhibited lower functional abundance in LCS_F compared to Control_F (Figure 8). In particular, a significantly reduced functional abundance of the substance dependence pathway was detected in LCS_F when compared to Control_F, Control_M, and LCS_M (χ^2^ = 13.853, df = 6, *p* = 0.031). Additionally, although no significant difference was found in the functional abundance of the substance dependence pathway across the three egg samples, their average richness followed the order: Egg_E > Egg_F1 > Egg_L (Supplementary Figure S3), consistent with their respective egg hatch rates.

## Discussion

Insect reproductive tracts harbor a myriad of endosymbiotic microbes, and Actinobacteria and Proteobacteria have been reported to be the most typical bacteria (Otti, 2015). Here we found that the most abundant bacteria in reproductive tracts of both cold-stored and non-stored control *H. axyridis* adults was Proteobacteria, followed by Firmicutes, Actinobacteria, and Bacteroidota. Our results aligned with previous studies conducted on the entire body of laboratory-reared *H. axyridis* adults (Du et al., 2022) as well as the hibernant adults from the invasive area of Poland (Dudek et al., 2017). However, despite observing similar bacterial abundance and richness across all four reproductive tract samples, long-term cold storage significantly altered the structure of the bacterial community within reproductive tracts of female beetles (LCS_F). At the phylum level, Proteobacteria obtained an increased relative abundance in LCS_F (79.64%) compared to Control_F (42.37%), while Firmicutes and Actinobacteriota displayed a decreased relative abundance in LCS_F. These alterations in the bacterial structure of cold-stored beetles may be partially attributed to low temperature-activated stress proteins or other physiological responses, which could potentially led to the elimination or reduction of certain bacterial species. For example, low temperature can induce an increased production of antioxidant enzymes in *H. axyridis* adults (Awad et al., 2021). However, it should be noted that a high activity of antioxidant enzymes may create an unfavorable environment for the growth of some specific bacteria, such as the intestinal microbiota in *Bombyx mori* L. (Shu et al., 2023). Functionally, these three aforementioned bacterial phyla have been demonstrated to play crucial roles in energy metabolism and nutrient absorption, thereby contributing to the successful growth and reproduction of their hosts (Schrempf, 2001; Reid et al., 2011; Flint et al., 2012; Colston and Jackson, 2016). Moreover, it has been reported that the ratio of Firmicutes to other bacteria is associated with some human diseases (Ley et al., 2006). Here, we deduced that alterations in the bacterial structure within the reproductive tracts may exert a significant impact on energy metabolism and nutrient absorption of LCS_F, consequently leading to various physiological disorders. Notably, there was a marked decrease in the functional abundance of the substance dependence pathway (a constituent of human diseases pathway at level 1) observed in LCS_F compared to Control_F. In Pacific white shrimp, *Litopenaeus vannamei* (Boone), it has been demonstrated that enhanced microbial functions associated with the substance dependence pathway play a crucial role in promoting growth when zymosan-A is included as part of their diet (Zheng et al., 2022). This finding validates the pivotal functions attributed to this particular pathway during host development.

Further, we observed a significant increase in the abundance of unclassified_f_Enterobacteriaceae within the phylum Proteobacteria in LCS_F (55.58%) as compared to Control_F (3.27%), which become the most representative bacterial genera in LCS_F. In line with our findings, Luo et al. (2023) reported an augmented prevalence of Enterobacteriaceae in *H. axyridis* adults subjected to pesticide pressure through predation on azadirachtin-treated *S. frugiperda* larvae. The authors suspected that Enterobacteriaceae might contribute to the dynamic equilibrium regulation of bacterial function (Luo et al., 2023). Additionally, previous studies have demonstrated the pivotal roles played by Enterobacteriaceae in digestion, protection, courtship, and reproduction (Ami et al., 2010; Hu et al., 2018; Zhao et al., 2018). Feeding *H. axyridis* with three different diets, Huang et al. (2022) observed a positive correlation between the increased abundance of Enterobacteriaceae and a decrease in pre-oviposition duration and an increase in egg production. However, our study revealed that cold-stored beetles exhibited a significant decline in the three-day average egg hatch rates, which reached nearly zero on days 17–19. Furthermore, we observed that eggs produced by F1 offspring of cold-stored beetles displayed severely reduced hatchability from the onset of oviposition, indicating transgenerational effects (Hackermann et al., 2008). The extent to which the marked increase in unclassified_f_Enterobacteriaceae within the female reproductive tract of cold-stored *H. axyridis* adults is associated with their poor reproductive performance remains unclear. Here, we deduced the existence of intricate interactions between bacteria and *H. axyridis* in relation to reproduction, as different bacterial species may exhibit contrasting functionalities. For example, Ju et al. (2020) found that *Wolbachia* infection significantly increased egg production in two planthopper species [*Laodelphax striatellus* (Fallén) and *Nilaparvata lugens* (Stål)] through vitamin Bs supplementation. However, Wang et al. (2023) observed a noteworthy reduction in female fecundity of phosphine-exposed *Tribolium castaneum* (Herbst) upon inoculation with *Enterococcus* sp., attributed to the suppression of expression and activity of host antioxidant enzymes. Furthermore, the functionality bestowed upon the host by a particular bacterium has been demonstrated to be environment-dependent (Guilhot et al., 2020).

Awad et al. (2023) have stressed that a below 60% egg hatchability of the laboratory-reared aphidophagous ladybirds should be of concern being affected by some factors causing non-fertilization or embryonic death. In this study, we further investigated the dynamic changes in the bacterial community within eggs produced by cold-stored beetles and their F1 offspring. No significant difference was observed in bacterial richness and diversity among Egg_E, Egg_L and Egg_F1. However, the changes in relative abundance of certain bacteria exhibited a consistent trend with changes in egg hatchability. For example, higher relative abundances of *Staphylococcus* and *Carnimonas* were detected in Egg_E compared to Egg_L and Egg_F1. *Staphylococcus* is known for producing proteases that facilitate protein absorption by their hosts (Collado et al., 2008; Carmen et al., 2010), which may play a crucial role in egg development and maturation (Coelho et al., 2016). In contrast, a higher abundance of *Buchnera* and *Serratia* was detected in Egg_L and Egg_F1 compared to Egg_E. *Buchnera* and *Serratia* are primary and secondary symbiotic bacteria widely found in *A. pisum*, but were found in *H. axyridis*, especially in adults (Du et al., 2022). These bacterial species are likely transmitted to the eggs primarily through adult consumption of *A. pisum* (Du et al., 2022; Hilker et al., 2023). It has been documented that *Serratia* in *A. pisum* can rapidly kill *H. axyridis* larvae, protecting them indirectly (Kovacs et al., 2017). Here, our findings revealed that the differences in bacterial community composition also align with the substance dependence pathway, following the richness order of Egg_E > Egg_F1 > Egg_L, which corresponds to the respective egg hatch rates. Combined with the decreased relative abundance in substance dependence pathway in LCS_F, it can be inferred that a dysfunction in this pathway, induced by an imbalanced bacterial community, may serve as one of the key factors contributing to egg inviability. However, more studies should be conducted to further reveal the mechanisms, such as investigating hatching rate of eggs normally laid after inoculation with the reproductive tract microorganisms of individuals after cold storage. In addition, a recent study has reported a significantly reduced hatchability (19%) in *H. axyridis* when the same female was co-infested with the fungus *Hesperomyces harmoniae* and the bacteria *Spiroplasma* compared to females infested with no or just one of the symbionts (59%) (Awad et al., 2023). Hence, further investigation is warranted to elucidate the role of reproductive tract microbial communities, including fungi, in adult *H. axyridis*.

## Conclusion

Cold storage is an influential technique to extend the shelf life of insects used as tools for biological control, and microbial structures should be of interest to shed light on the mechanisms by which long-term cold storage affects host insect quality. The present investigation provides a comprehensive description of alterations in bacterial communities within the reproductive tracts of cold-stored *H. axyridis* adults. We detected a remarkable increase in unclassified_f_Enterobacteriaceae within the female reproductive tract of LCS_F. Moreover, variations were observed in relative abundance of certain bacterial genera in eggs produced by cold-stored beetles and their F1 offspring, which positively correlated with changes in egg hatch rates. Finally, based on functional prediction, we have identified a potential deficiency in the substance dependence pathway as one of key factors contributing to the reduced or lost egg hatchability in cold-stored beetles as well as in their offspring. Our findings might be the first report investigating changes in bacteria within the reproductive tracts of cold-stored insects used as tools for biological control and exploring potential correlations between bacterial alterations and changes in egg hatchability. These results may broaden our knowledge in understanding the bacteria-host interactions under environmental pressures.

## Data availability statement

The datasets presented in this study can be found in online repositories. The names of the repository/repositories and accession number(s) can be found below: PRJNA1000108.

## Ethics statement

The manuscript presents research on animals that do not require ethical approval for their study.

## Author contributions

YS: Supervision, Writing – review & editing. YH: Methodology, Writing – original draft. SW: Supervision, Writing – review & editing. XC: Methodology, Writing – original draft.

## Supplementary material

SUPPLEMENTARY figure S1 Alpha diversity indices of microbial community. **(A)** Coverage index. **(B)** Shannon index. **(C)** Chao index. A sterisk indicates a significant difference in the pairwise comparison of the two groups (*p* < 0.05).

SUPPLEMENTARY FIGURE S2 Linear discriminant analysis effect size (LEfSe) analysis of microbial abundance of Egg_E, Egg_L and Egg_F1. **(A)** Linear discriminant analysis effect size. **(B)** Taxonomic cladogram derived from LEfSe with an LDA score > 2.0.

SUPPLEMENTARY FIGURE S3 Relative abundance of microbial species involved in substance dependence pathway at level 2. Asterisk represents a significant difference in the pairwise comparison of the two groups (*p* < 0.05).
