# Supplementary figures and images for: Changes in the bacterial communities of Harmonia axyridis (Coleoptera: Coccinellidae) in response to long-term cold storage and progressive loss of egg viability in cold-stored beetles
Source: Front Microbiol. 2024 Mar 12;15:1276668. doi: 10.3389/fmicb.2024.1276668 (PMC10964723; doi:10.3389/fmicb.2024.1276668)

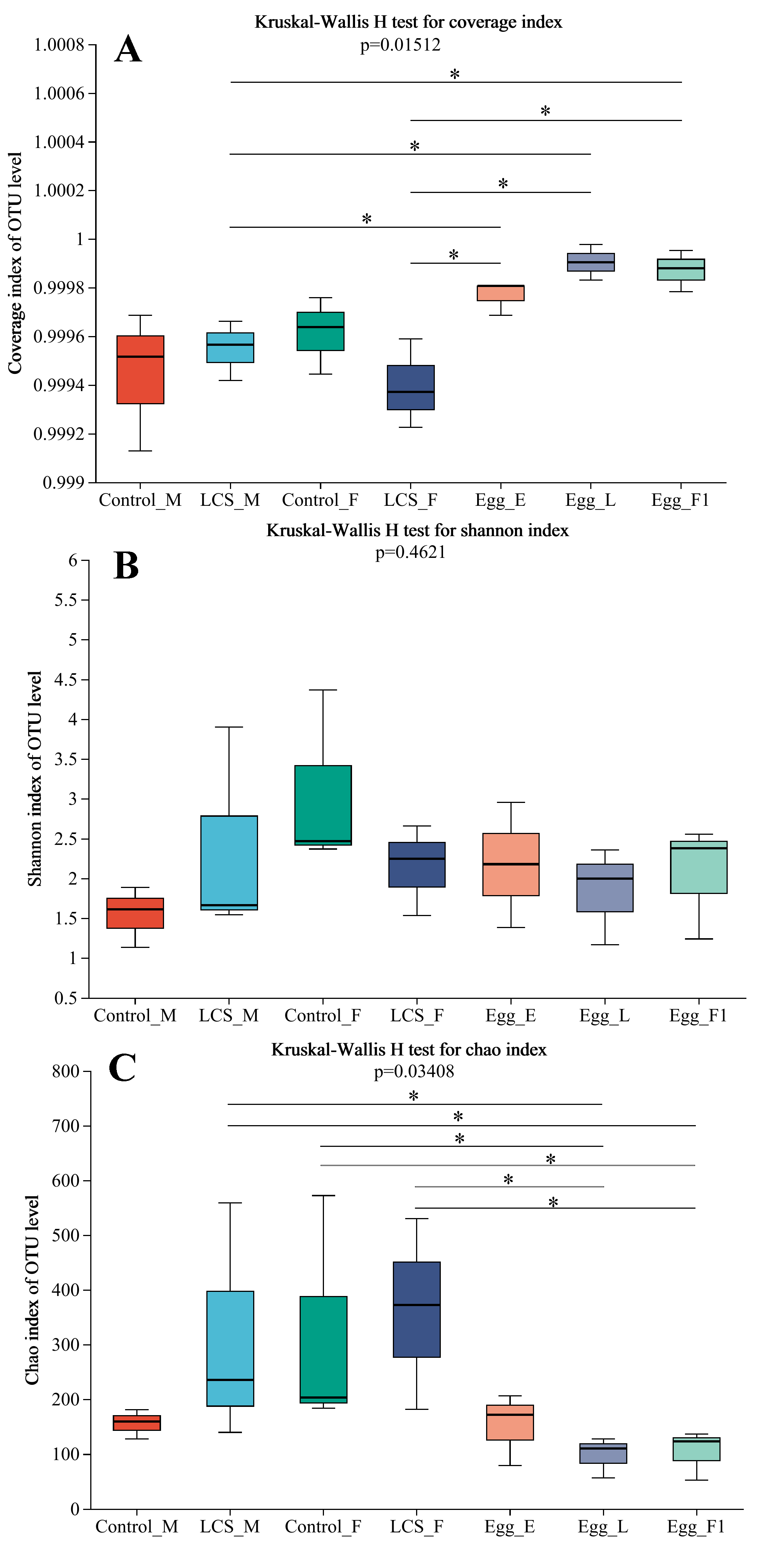

Supplement: SUPPLEMENTARY figure S1 — Alpha diversity indices of microbial community. (A) Coverage index. (B) Shannon index. (C) Chao index. A sterisk indicates a significant difference in the pairwise comparison of the two groups (p < 0.05). [file Image_1.tif]

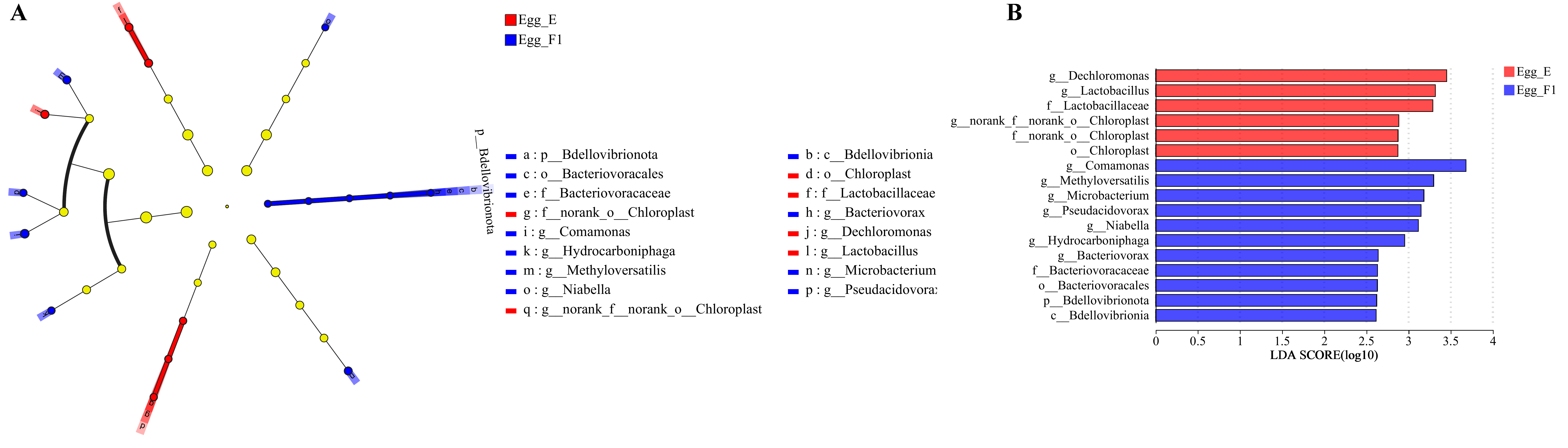

Supplement: SUPPLEMENTARY FIGURE S2 — Linear discriminant analysis effect size (LEfSe) analysis of microbial abundance of Egg_E, Egg_L and Egg_F1. (A) Linear discriminant analysis effect size. (B) Taxonomic cladogram derived from LEfSe with an LDA score > 2.0. [file Image_2.tif]

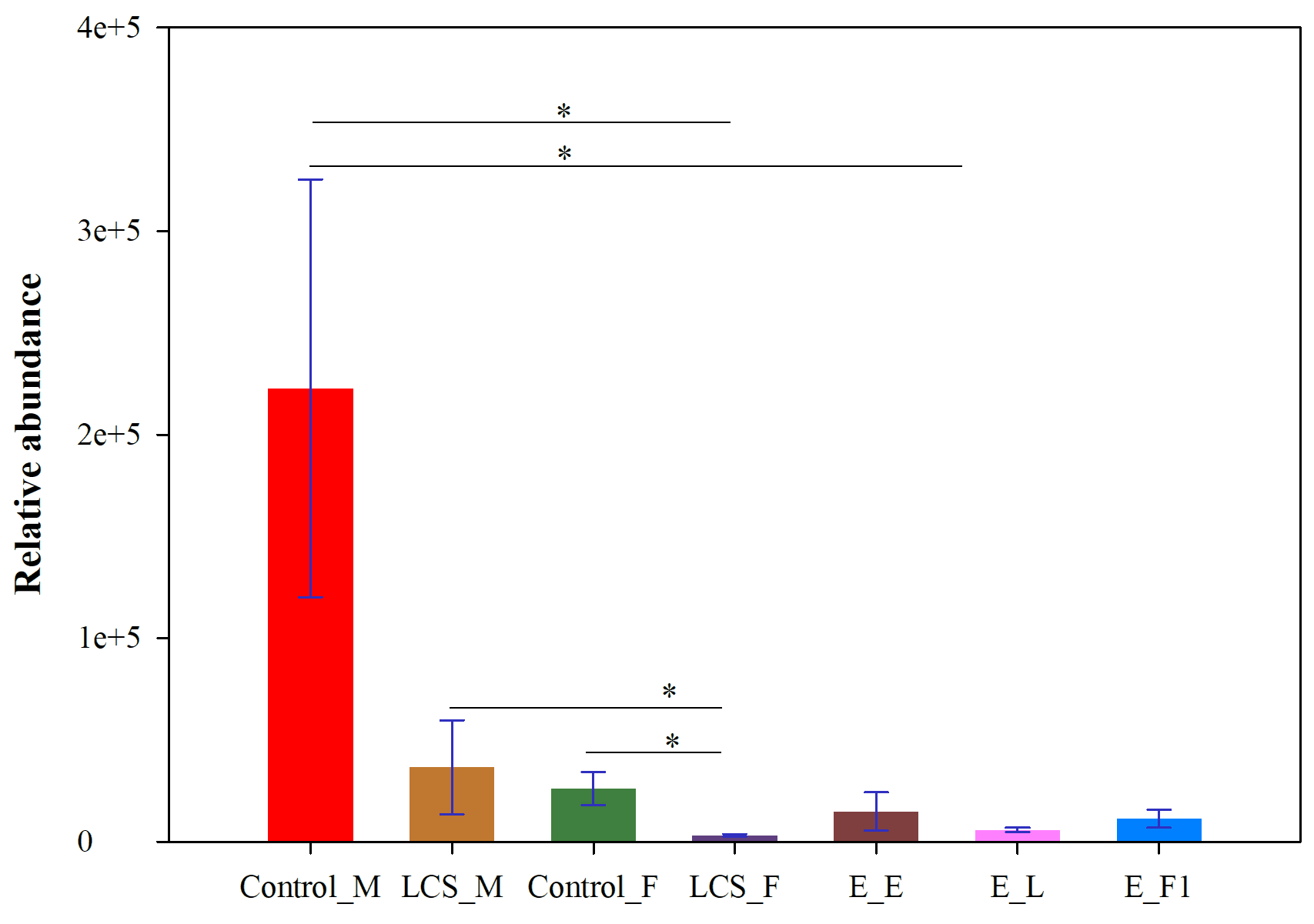

Supplement: SUPPLEMENTARY FIGURE S3 — Relative abundance of microbial species involved in substance dependence pathway at level 2. Asterisk represents a significant difference in the pairwise comparison of the two groups (p < 0.05). [file Image_3.tif]
